# Network analysis of microRNA and mRNA seasonal dynamics in a highly plastic sensorimotor neural circuit

**DOI:** 10.1186/s12864-015-2175-z

**Published:** 2015-11-06

**Authors:** Tracy A. Larson, Karin L. Lent, Theo K. Bammler, James W. MacDonald, William E. Wood, Melissa L. Caras, Nivretta M. Thatra, Agata Budzillo, David J. Perkel, Eliot A. Brenowitz

**Affiliations:** Department of Biology, University of Washington, Seattle, WA 98195 USA; Present Address: Basic Sciences Division, Fred Hutchinson Cancer Research Center, 1100 Fairview Ave N, Seattle, WA 98109 USA; Department of Psychology, University of Washington, Seattle, WA 98195 USA; Department of Environmental and Occupational Health Sciences, University of Washington, Seattle, WA 98195 USA; Department of Otolaryngology, University of Washington, Seattle, WA 98195 USA; Graduate Program in Neuroscience, University of Washington, Seattle, WA 98195 USA; Present address: Centre National de la Recherche Scientifique, Laboratoire de Neurophysique et Physiologie, UMR 8119, Université Paris Descartes, 45, rue des Saints Pères, 75006 Paris, France; Present address: Center for Neural Science, New York University, 4 Washington Place, New York, NY 10003 USA

**Keywords:** microRNA, miR–mRNA network, Adult neurogenesis, Seasonal plasticity, Sex steroids, Testosterone, Photoperiod, Songbird, Bird song

## Abstract

**Background:**

Adult neurogenesis and the incorporation of adult-born neurons into functional circuits requires precise spatiotemporal coordination across molecular networks regulating a wide array of processes, including cell proliferation, apoptosis, neurotrophin signaling, and electrical activity. MicroRNAs (miRs) - short, non-coding RNA sequences that alter gene expression by post-transcriptional inhibition or degradation of mRNA sequences - may be involved in the global coordination of such diverse biological processes. To test the hypothesis that miRs related to adult neurogenesis and related cellular processes are functionally regulated in the nuclei of the avian song control circuit, we used microarray analyses to quantify changes in expression of miRs and predicted target mRNAs in the telencephalic nuclei HVC, the robust nucleus of arcopallium (RA), and the basal ganglia homologue Area X in breeding and nonbreeding Gambel’s white-crowned sparrows (*Zonotrichia leucophrys gambelli*).

**Results:**

We identified 46 different miRs that were differentially expressed across seasons in the song nuclei. miR-132 and miR-210 showed the highest differential expression in HVC and Area X, respectively. Analyzing predicted mRNA targets of miR-132 identified 33 candidate target genes that regulate processes including cell cycle control, calcium signaling, and neuregulin signaling in HVC. Likewise, miR-210 was predicted to target 14 mRNAs differentially expressed across seasons that regulate serotonin, GABA, and dopamine receptor signaling and inflammation.

**Conclusions:**

Our results identify potential miR–mRNA regulatory networks related to adult neurogenesis and provide opportunities to discover novel genetic control of the diverse biological processes and factors related to the functional incorporation of new neurons to the adult brain.

**Electronic supplementary material:**

The online version of this article (doi:10.1186/s12864-015-2175-z) contains supplementary material, which is available to authorized users.

## Background

Ongoing neurogenesis in the adult brain requires complex but yet precise temporal and spatial coordination of the underlying processes and mechanisms. For example, neural stem cells residing in specific niches throughout the adult brain [1–4] proliferate and give rise to new neurons and glia [5]. The new immature neurons depart the neurogenic niche, migrate to their final destinations [5, 6], and integrate into functional circuits [5, 7–9]. Once fully integrated mature adult-born neurons persist for periods ranging from days to years [5, 10–12]. Each of these processes is regulated by a plethora of interacting autonomous and non-autonomous factors. Some of these factors include but are not limited to sex steroid hormones secreted by the gonads and synthesized *de novo* in the brain, locally synthesized neurotrophins, neural use and activity, cell death and inflammation, behavior including social interactions, and stress (for reviews see [5, 13]).

One potential candidate for globally regulating the different biological processes, mechanisms, and factors associated with adult neurogenesis is microRNAs (miRs). miRs are short, non-coding RNA sequences that alter gene expression by translational repression or mRNA target degradation (for review see [14]). Individual miRs have many mRNA targets, and thus can act as global regulators of complex temporal and spatial patterns of gene or protein expression changes underlying neural plasticity [14]. Moreover, miR expression is highly enriched in the brain [15] and has been implicated as involved in a variety of neurological disorders and diseases including Amyotrophic Lateral Sclerosis [16], Fragile X mental retardation [17], mood and mental disorders [18, 19], and Alzheimer’s Disease [20]. Specific miRs play major roles in the normal processes of neural plasticity including fate specification [21], dendritic arborization and synapse formation [22, 23], adult-born neuronal addition and survival [24], and apoptosis [25]. However, potential genetic regulatory networks of brain-expressed miRs have been little explored in the context of adult neural circuit plasticity.

**Fig. 1:**
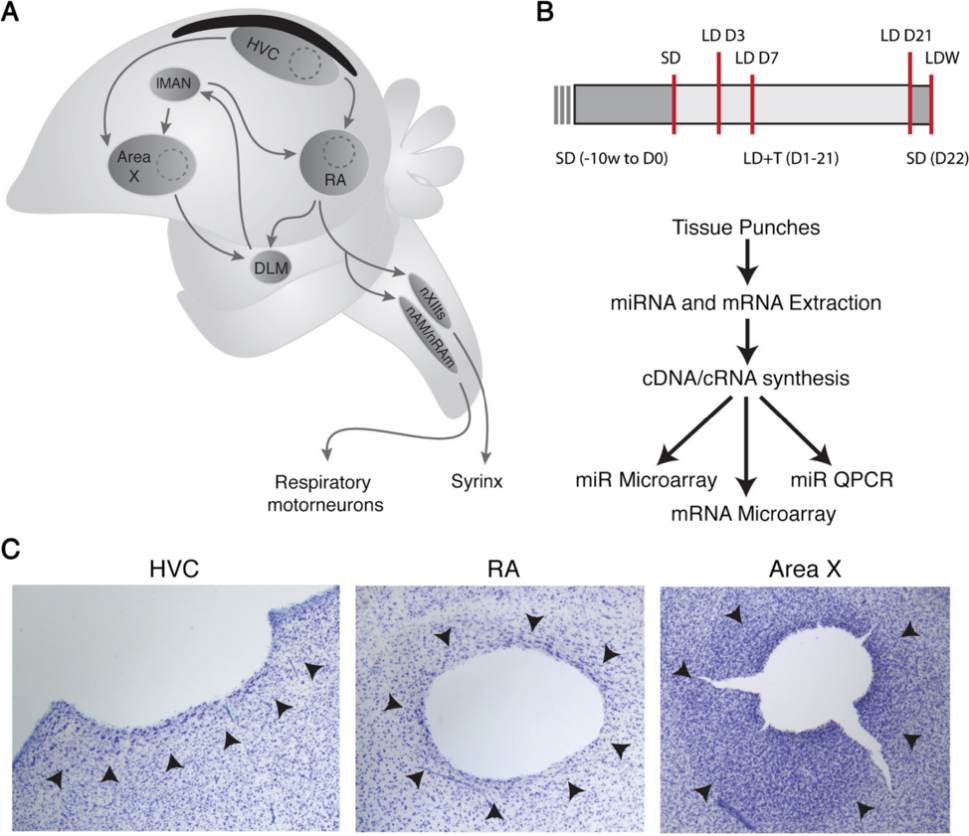
Experimental design. **a** A schematic of the song nuclei sampled for microarray analysis. The dotted line indicates where tissue samples were obtained. **b** Experimental time-line for all experimental groups. A red line indicates termination of the experiment for the given group. **c** Representative images of Nissl-stained brain sections confirming tissue punch locations in HVC, RA and Area X. The arrowheads indicate the borders of the respective nuclei as determined by cell morphology and density

One prominent model for adult neurogenesis is the song control circuit of songbirds (Fig. 1a). Adult neurogenesis in Area X, a basal ganglia homologue required for song learning [26], occurs at high constitutive rates [27]. On the other hand, adult neurogenesis in HVC, a pallial nucleus involved in song learning and production, exhibits pronounced seasonal changes in neuronal addition and neuronal loss (reviewed in [13]). Most, if not all, of the new neurons added to the adult HVC have long axons that project 4 mm or more to synapse on target cells in RA [28, 29]. During the breeding season total neuronal number in HVC of Gambel’s white-crowned sparrow (*Zonotrichia leucophrys gambelii*) increases by 25 % (nearly 68,000 neurons), due to increased addition of adult-born neurons [30–32]. As white-crowned sparrows transition back into nonbreeding conditions, HVC neuronal number decreases through caspase-mediated apoptosis of neurons [33–35]. The seasonal incorporation of new HVC neurons of adult birds, correlates with changes in song production - a learned sensorimotor behavior; each breeding season as HVC increases in neuronal number, song production rate and song stereotypy increase [28, 36, 37]. Concomitant with the changes in HVC morphology and singing behavior, both Area X and RA change in morphology [27, 32, 33] and RA in neuronal activity during breeding seasons [38, 39]. The quantitative changes in HVC neuronal number, the incorporation of adult-born neurons into long-range neural circuits, and the tight relationship between HVC adult neurogenesis, morphological and physiological changes in other song nuclei, and the production of a learned sensorimotor behavior, make the HVC ➝ RA and HVC ➝ Area X circuits a unique model for investigating the spatiotemporal pattern of genetic networks that regulate the multitude of processes and factors related to adult neural circuit plasticity.

## Methods

### Animals

All animal procedures were approved by the Institutional Animal Care and Use Committee at the University of Washington. Sixty adult male Gambel’s white-crowned sparrows (*Zonotrichia leucophrys gambelii)* were collected in eastern Washington during their spring and autumnal migration under State of WA Scientific Collecting permit #10-162 and U.S. Fish and Wildlife Permit #MB708576-0. Birds were housed in outdoor aviaries under natural photoperiods for at least 20 weeks prior to transitioning into indoor aviaries. Once indoors, birds were exposed to a short-day photoperiod (SD; 8 h light: 16 h dark) for at least 10 weeks prior to experiment onset to ensure that they were photosensitive and responsive to sex steroid hormones. Food and water were available *ad libitum* throughout the experiment. We castrated all birds by anesthetizing them with isoflurane, making a small incision on the left side anterior to the caudal-most rib and dorsal to the uncinate process, and aspirating both testes [40].

To synchronize the physiological states of the birds, birds were implanted with a subcutaneous Silastic pellet (i.d. 1.0 mm; o.d. 2.0 mm; length: 12 mm; VWR) filled with crystaline T (Sigma) and shifted to a long day photoperiod (LD; 20 h light: 4 h dark) for 21 days (see Fig. 1b for experimental design). A period of 21 days in breeding-like conditions is adequate for full breeding-like growth of the song circuits [32]. On day 21 we removed the subcutaneous T pellets from all birds, and shifted them back to SD photoperiods for 10 weeks. On the final day of SD we quickly decapitated nine birds and removed the brain for processing as detailed below. The SD group of birds represented the steady-state regressed song control circuit and served as a baseline of comparison for all other groups. Another group of 45 birds were transitioned back to LD photoperiods and implanted with T (LD + T). On days 3, 7, and 21 of LD + T exposure, we quickly decapitated nine birds from each group and removed the brain. Of the nine remaining birds in LD + T for 21 days, all had T pellets removed and were transitioned back to SD overnight (i.e. LDW condition). After 1 day in SD, all nine remaining birds were killed for tissue collection. All birds were allowed to sing throughout the course of the entire experiment. SD and LDW birds sing less stereotyped song and do so less frequently than LD + T birds [37]. There is extensive variability in song rate within and between individual birds over a given day, and from day to day, under LD + T conditions [41]. To control for circadian effects on genes and behavior, we killed all birds between 4 and 8 h after lights on.

### Tissue harvesting

The brains of all birds were removed rapidly (within 1 min) and Vibratome sectioned at 300 μm in ice-cold, oxygenated artificial cerebral spinal fluid (ACSF; 119 mM NaCl, 2.5 mM KCl, 1.3 mM MgSO4, 2.5 mM CaCl2, 1 mM NaH2PO4, 16.2 mM NaHCO3, 11 mM D-glucose, and 10 mM HEPES). From these slices, punches of tissue containing HVC, RA, or Area X were collected within 2 min under a dissecting microscope using blunted hypodermic needles of the minimum gage that could fit entirely within the target nucleus. Tissue punch location was verified post-hoc by Nissl-staining of re-sectioned, fixed tissue, as in (Fig. 1c; [42]). HVC punches included the proliferative ventricular zone just dorsal to HVC. All of tissue punches from one nucleus of one bird were pooled, flash frozen in dry ice, and stored at –80 °C until processed for microarray hybridization.

### RNA isolation to microarray hybridization

Total RNA was extracted from individual snap frozen tissue samples using the mirVana Paris Kit according to the manufacturer’s protocol (Life Technologies, PN 1556 M Rev C) to generate samples of isolated small (<200 nucleotides) and large (>200 nucleotides) RNAs. Total RNA concentration was determined measuring OD260, and the integrity of each RNA sample was verified using an Agilent 2100 Bioanalyzer (Santa Clara, CA). Only RNA samples with appropriate size distribution, quantity, and OD260/280 and OD 260/230 ratios of 1.8–2.1 were used for further array analysis. Total RNA samples were used for mRNA and microRNA arrays analysis.

For mRNA analysis, samples were processed and hybridized to Agilent ZebraFinch Oligoarray v2.2 (Agilent, Santa Clara, CA). The Agilent ZebraFinch Oligoarray v2.2 was designed with 43,488 60-mer oligos representing 10,647 annotated genes ([43, 44]). RNA isolation from brain punches resulted in a lower yield than is required for Agilent ZebraFinch Oligoarray v2.2. Therefore, RNA amplification was performed using the Nugen Ovation PicoSL WTA kit according to the manufacturer’s protocol. Amplified RNA was used for further processing and hybridization to the Agilent arrays using the manufacturer’s established one color protocol. Hybridization and washing of these arrays was accomplished using HS 400 Pro hybridization and wash stations (Tecan Systems, Inc., San Jose, CA). In total 45 microarrays were used, including three replicates for each nucleus from each experimental time point. Arrays were scanned using an Agilent DNA Microarray Scanner (Agilent Technologies, Inc. Santa Clara, CA), according to the manufacturer’s established standard protocol.

For miR analysis, samples were processed and hybridized to Affymetrix miRNA 3.0 arrays (Affymetrix, Santa Clara, CA). The Affymetrix miRNA 3.0 array was designed based on the miRBase v17 database. This array contains probe sets for 19,724 mature microRNAs from a total of 153 species including several avian species. Samples isolated form three individual birds belonging to the same experimental groups were pooled. For each experimental group, three biological replicates of pooled samples (i.e., a total of nine birds in three pooled samples) were processed and hybridized to Affymetrix miRNA 3.0 arrays according to the manufacturer’s recommended protocol. Arrays were scanned with an Affymetrix GeneChip® 3000 scanner. In total 45 microarrays were used, including three replicates for each nucleus from each experimental time point. The hybridized arrays were scanned with an Affymetrix GeneChip 3000 scanner.

### Microarray data analysis

Raw mRNA array data from the Agilent Zebrafinch Oligoarrays was extracted with the Agilent Feature Extraction image analysis software (Agilent, Santa Clara, CA). The data were normalized using a variance-stabilization procedure (VSN) [45]. Image generation and feature extraction for Affymetrix microRNA 3.0 arrays was performed using Affymetrix GeneChip Command Console Software. The raw microRNA array data was normalized using quantile normalization, followed by a robust multi-array average (RMA; [46]) with Bioconductor [47]. Several quality control steps were followed: (1) visual inspection of the GCOS chip images, (2) visual inspection of the chip pseudoimages generated by the Bioconductor affyPLM package, (3) generation and inspection of principal components analysis (PCA) plots, (4) generation and inspection of histograms of raw signal intensities, and (5) generation and comparison of the Relative Log Expression and Normalized Unscaled Standard Errors using the Bioconductor affyPLM package. MiRs and mRNAs with significant differential expression were identified using the Bioconductor limma package [48]. Data were analyzed using a weighted analysis of variance (ANOVA) model, making individual comparisons using empirical Bayes adjusted contrasts. The weighted ANOVA model assigned array weights to smoothly up or down-weight the importance of a particular array, based on how similar that array is to others of the same type [49]. The empirical Bayes adjustment estimates a variance prior based on all genes or miRNAs on the array, and then reduces the by-gene estimates towards that prior [48].

We compared the differentially expressed mRNAs from our study to previously published gene expression data sets ([40, 44]; GSE28347 and GSE33365, respectively) using R software (http://www.r-project.org/). We filtered both gene expression data sets for differentially expressed genes using the published filtering criteria used in the prior studies (i.e. a threshold of 1.5-fold change and *p* < 0.05 for the Thompson et al. (2012) data, and a 2-fold change and *p* < 0.01 for Whitney et al. (2014); [40, 44]). Prior to making comparisons we assigned HUGO Gene Nomenclature Committee symbols (http://www.genenames.org) to the probes. This resulted in a 75 % overlap in gene symbols between our array targets and the targets of the arrays used in Thomspon et al. (2012). Whitney et al. (2014) utilized the same array platform as we did. We compared the differentially expressed genes in HVC, RA, and Area X in LD + T 3, 7, and 21D and LDW relative to SD across our and the Thompson et al. (2012) datasets. We compared our results from LD + T 21D relative to SD to the differentially expressed genes between 5 h of singing and no singing of Whitney et al. (2014).

### Real-time quantitative PCR confirmation of miR expression

Differentially expressed genes of interest were selected for internal validation of the microarray results by TaqMan based real-time quantitative reverse transcription (qRT-PCR). The goal of the qRT-PCR was to confirm the identity and expression of genes shown by the microarray analysis to be differentially expressed. Four micro-liters of remaining cRNA not used for microarray hybridization was added to each 25 μl PCR mixture consisting of primers (0.16 μM each) specific to one miR of interest, buffers, salts, and SYBR Green PCR master mix. Fluorescence detection was measured using the 7900HT FAST Real-Time PCR System (Applied Biosystems, Foster City, CA) with the following PCR reaction profile: 1 cycle of 95 °C for 10 min, 40 cycles of 95 °C for 30 s, and 60 °C for 60 s, followed by a melt curve. DNA amplification was quantified from the *C* (*T*) value based on standard curves to ensure quantification was within a linear range. All signals were normalized against U6, and fold-change ratios were calculated for experimental samples compared to SD controls using R software. miR-132 qRT-PCR expression across nuclei at LD + T 3D, 7D, 21D, and LDW relative to SD were fit with ANOVA and had p-values adjusted using a family-wise error rate (FWER) adjustment and subsequent Hommel correction for multiple comparison.

### MiR sequencing and analysis

In order to cross correlate seasonally regulated mRNAs to the differentially regulated miRs, we used the microRNA Target Filter function of the Ingenuity Pathway Analysis software (IPA, http://www.ingenuity.com) as described below. The miR sequence, specifically the mature and seed region sequences, were confirmed to be 100 % homologous to the equivalent human miR. Briefly, primers for PCR were designed to amplify the full sequence of Gambel’s white-crowned sparrow miRs of interest using the sequence of that miR in closely related bird species and a broad range of vertebrates. From genomic DNA, full genomic miR sequences were amplified with PCR using Quick Load Master Mix (New England Biolabs) as per the manufacturer’s protocol. MiR sequence was verified (via GeneWiz, Seattle, WA) from independent PCR product of five individual birds after isolation from a 1.5 % electrophoresis gel using the Qiagen Gel Extraction Kit. All miRs of interest were confirmed to have 100 % identity within the mature and seed regions of human and mouse sequences (Additional file 1: Table S1). For miR-132 and miR-210, rooted phylogenetic trees with branch lengths were constructed in ClustalW (www.genome.jp/tools/clustalw/) using the white-crowned sparrow sequence and the respective miR sequences of other vertebrates obtained through the miRBase, v17 database (mirbase.org, University of Manchester) or when not available, as with miR-210, annotated sequences from bird (taxid:8782) BLAST hits (blast.ncbi.nlm.nih.gov; Additional file 2: Figures S1 and Additional file 3: Figure S2).

### Canonical signaling pathway and miR target predictions

Conserved canonical signaling pathways were predicted for both mRNAs and miRs using the core analysis module of the IPA software (Ingenuity, http://www.ingenuity.com). Canonical pathways with *p* < 0.05 were considered significant. Potential targets of miR-132 and miR-210 were predicted using the miR Target Filter module in IPA (Ingenuity) and applying the filtering criteria moderate or high confidence based on the TargetScan database, as well as experimentally validated targets based on the miRBase and miRecords databases. It is important to recognize that the IPA data base contains mRNA sequence information from mouse, rat, and human, but not white-crowned sparrow or other avian species. Therefore, the IPA predicted microRNA targets are based on these mammalian mRNA sequences. Currently a white-crowned sparrow data base is not available to identify predicted targets of microRNAs. We used the following approach to minimize the chance of drawing conclusions based on incorrectly predicted targets. First, we performed pathway analyses with two miRs – miR-132 and miR-210-both of which have 100 % identity in white-crowned sparrows with the respective human miR sequence. Second, we overlaid and filtered all miR targets with the white-crowned sparrow mRNA array data generated using the same samples with the IPA predicted targets. We only used those predicted mRNA targets that were also inversely differentially expressed (>1.5-fold, *p* < 0.005) in our white-crowned sparrow mRNA array data for further analysis. Third, we focused our analysis on identified pathways suggested by microRNA targets, thereby minimizing the potential pitfall of placing too much emphasis on single target genes.

## Results and discussion

### Seasonal expression of mRNAs in the song control nuclei

**Fig. 2:**
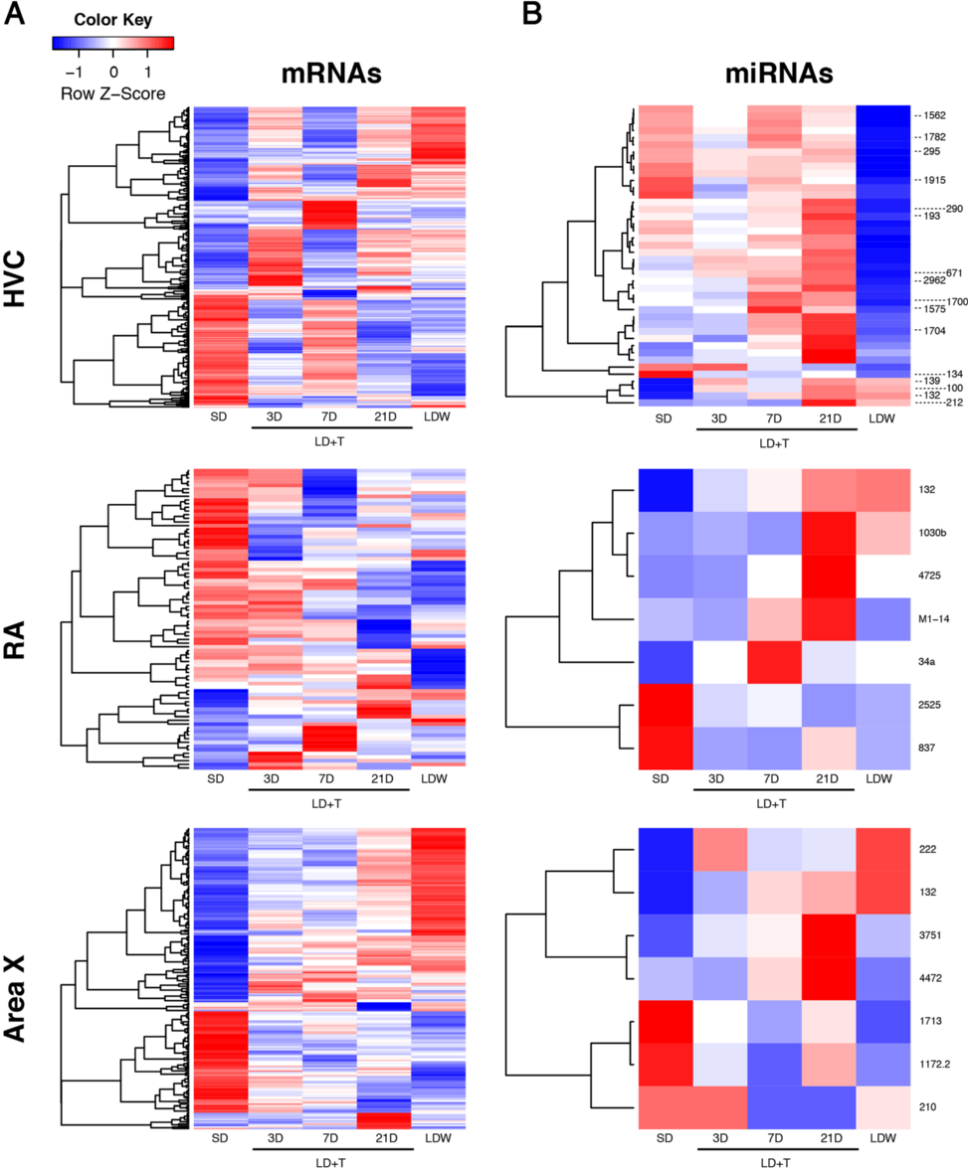
Patterns of changing expression of mRNA and miR transcripts between breeding and nonbreeding conditions. Expression data were converted to z-scores for each mRNA or miR. Thus colors represent up (red) or down (blue) regulation of a miR or mRNA in a particular sample, as compared to the mean expression for that gene. **a** The relative expression of mRNAs that were differentially regulated in at least one experimental group (i.e. LD + T 3D, 7D, 21, or LDW) compared to SD. A *p*-value of <0.0001 and a fold change >2.0 were used as the threshold for inclusion. **b** Relative expression of miRs that were differentially regulated in at least one experimental group with a fold change >2.0 and *p* < 0.0005 compared to SD. miRs of interest based on predicted function or miRs specific to birds are denoted

We identified differential expression of mRNA between breeding and nonbreeding condition using microarray analyses of tissue harvested from HVC, RA, and Area X, in SD, LD + T at 3D, 7D, 21D, and LDW conditions. We obtained 155, 61, 43, and 67 of differentially expressed (fold >2.0, *p* < 0.0001) mRNAs in HVC at LD + T 3D, 7D, 21D, and LDW, respectively (Fig. 2a; Additional file 4: Table S2). In RA 7, 14, 10, and 10 and in Area X 21, 25, 29, and 81 genes varied by >2.0 fold change with *p* < 0.0001 in LD + T 3D, 7D, 21D, and LDW, respectively (Fig. 2a; Additional file 4: Table S2). We found in the neurogenic nuclei HVC and Area X that only 8.6–13.9 % and 31.0–41.9 %, respectively, of the genes that were differentially expressed across all time points were down regulated (Fig. 2a; Additional file 4: Table S2). In the non-neurogenic nucleus RA we found a higher percentage of genes down-regulated and greater variability in this percentage across experimental groups compared to the neurogenic nuclei; the percentage of down regulated genes in RA varied from 50 to 97 % across all time points. We used IPA to identify significant pathways in our data. Seasonally regulated mRNAs were involved in several canonical signaling pathways including ERK5 and NGF signaling in HVC and WNT/Ca + and axonal guidance signaling in RA (Table 1). Upon transition from breeding to nonbreeding conditions (LDW group), we found that genes in the canonical signaling pathways for cell death (i.e. tumorcidal) and inflammation (i.e. LPS/IL-1) were differentially expressed compared to SD in HVC and Area X (Table 1).

**Table 1:** Top canonical pathways of mRNAs seasonally regulated in HVC, RA, and Area X

	Top Canonical Pathways	*p*-value	Ratio	# of Genes
HVC				
3D	RAN Signaling	0.0009	0.214	3
	Cell Cycle Control	0.0235	0.118	2
7D	Galactose Degradation	0.0280	0.200	1
	UDP-N-acetyl-D-galactosamine Biosynthesis	0.0057	0.143	1
21D	ERK5 Signaling	0.0001	0.103	4
	NGF Signaling	0.0064	0.062	5
LDW	ATM Signaling	0.0168	0.075	3
	Tumoricidal Function	0.0171	0.133	2
RA				
3D	Wnt/Ca + Pathway	0.0500	0.214	1
	Axonal Guidance Signaling	0.0500	0.008	2
7D	Myo-inositol Signaling	0.0081	0.086	3
	T Lymphcyte Signaling	0.0478	0.042	1
21D	Cancer Signaling	0.0027	0.056	2
	Synaptic Long Term Depression	0.0105	0.023	2
LDW	Sphingosine Metabolism	0.0054	0.500	1
	Ceramide Degradation	0.0054	0.500	1
Area X				
3D	Galactose Degradation	0.0152	0.200	1
	RAN Signaling	0.0420	0.071	1
7D	Flavin Biosynthesis	0.0029	1.000	1
	Salvage Pathway	0.0110	0.025	1
21D	Granulocyte Adhesion	0.0130	0.045	2
	Gylcerol Degradation	0.0197	0.200	1
LDW	LPS/ IL-1 Mediated Function	0.0060	0.056	5
	MIF-mediated Glucocorticoid Regulation	0.0100	0.167	2

Gene regulatory networks that control patterning and plasticity within song control nuclei have been previously examined with sequencing analysis of the zebra finch genome as well as microarray analysis [43, 50]. More specifically, Thompson et al. (2012) identified 132 genes in HVC cells that changed in expression between breeding and nonbreeding conditions when compared to gene expression in RA, a non-neurogenic region of the song bird brain [40]. By comparison, we identified 265 genes differentially expressed between HVC and RA in breeding conditions and 239 in nonbreeding conditions. In both studies, genes that promoted proliferation, angiogenesis, and neurite extension were up-regulated, whereas genes that support programmed cell death were down-regulated in HVC under breeding conditions [40]. Specific genes that encode neurotrophins known to promote neuronal migration, recruitment, and survival, including brain-derived neurotrophic factor (*BDNF*) and insulin-like growth factor 1 were differentially regulated in HVC under breeding conditions in both studies [40]. A detailed comparison of specific genes differentially regulated in both the current study and the data from Thompson et al. (2012) revealed that neuropeptide Y(*NPY*) was differentially expressed in HVC during LD + T 21D. No other genes were commonly shared as significantly differentially regulated across brain regions and across common experimental groups. This comparison was limited, however, by a 27 % concordance for clone IDs and 75 % concordance for gene symbols between the two array platforms used within these studies [40].

During breeding conditions, the period in which HVC incorporates a significant number or adult-born neurons, white-crowned sparrows sing with greater stereotypy and more often when compared to both SD and LDW [35, 37]. Previous studies have examined differential gene expression related specifically to singing behavior in zebra finches [44, 51]. For example, Whitney et al. (2014) identified 5167 differentially expressed transcripts in HVC, RA, Area X and LMAN that grouped into four superclusters of transient early and late-response increases and decreases. Examination of enriched gene pathways in both our study and Whitney et al. (2014) identified pathways related to MEF/ERK signaling in HVC, Wnt signaling in RA, and biogenesis in Area X [44]. Direct comparison of the differentially expressed genes in the LD + T 21D group of the white-crowned sparrows with those of zebra finches singing for 5 h versus no singing resulted in only one shared differentially expressed gene – *ASAP3* in Area X.

### miRs are differentially expressed in song control nuclei between breeding and nonbreeding conditions

To test whether miRs were differentially expressed between breeding and nonbreeding conditions, we quantified with microarray the fold-change relative to SD of over 150 unique miRs in tissue from HVC, RA and Area X of birds in LD + T and LDW. Of the three miRs that were significantly differentially expressed across all conditions (>2.0 fold change and false discovery rate = 0.05), miR-132, specifically within HVC, exhibited the highest fold change in expression (Additional file 5: Table S3). Using a less stringent threshold of fold change >2.0 and *p* < 0.0005, we obtained 4, 13, 19, and 29 differentially expressed miRs in HVC, at LD + T 3D, 7D, 21D, and LDW, respectively (Table 2 and Fig. 2b). In RA we found thirty miRs that were differentially expressed in one or more experimental groups, whereas in Area X only five miRs were differentially expressed with the fold change >2.0 and *p* < 0.0005 (Table 2 and Fig. 2b). The expression landscapes in HVC, RA, and Area X suggest that the majority of miRs are generally up-regulated with time into breeding condition (Fig. 2b). Concomitantly, large clusters of mRNAs became down-regulated with progression into breeding condition in all three nuclei (Fig. 2a). Alternatively, during LDW 13 miRs in HVC decreased significantly in expression compared to SD, while large clusters of mRNAs related to macrophage function (e.g. *MPEG1*), cell arrest (e.g. *ZAR1*), and sirtuin signaling (e.g. *SIRT3*) increased in expression.

**Table 2:** Significantly differentially expressed miRNAs with a fold change >1.5 and *p* < 0.0005

		3D			7D			21D			LDW	
HVC	miRNA	Fold Change	*p*-value	miRNA	Fold Change	*p*-value	miRNA	Fold Change	*p*-value	miRNA	Fold Change	*p*-value
	miR-132 (4)^a^	1.88 ± 0.18	0.0015	miR-132 (12)	2.83 ± 0.06	0.0033	miR-132 (13)	3.63 ± 0.07	<0.0001	miR-132 (13)	3.65 ± 0.10	0.0001
							miR-1356	2.00	<0.0001	miR-134	−1.12	0.0001
							miR-212	1.96	0.0001	miR-1182	−1.81	0.0005
							miR-2840	1.69	0.0004	miR-404	−1.83	0.0005
										miR-1915	−1.98	0.0002
										miR-395f	−2.11	0.0003
										miR-2455	−2.23	0.0003
										miR-1562	−2.39	0.0002
										miR-4516	−2.44	<0.0001
										miR-574	−2.45	0.0005
										miR-295	−2.53	0.0004
										miR-395b	−2.57	0.0001
										miR-1782	−2.68	0.0004
										miR-1362	−2.79	0.0001
RA												
	miR-281	4.47	0.0001	miR-34a	5.00	<0.0001	miR-132 (4)	1.88 ± 0.05	0.0001	miR-132 (4)	1.90 ± 0.11	0.0001
	miR-1192	3.98	0.0003	miR-2111n	4.84	<0.0001	miR-2525	−2.40	0.0004			
	miR-184	3.94	0.0004	miR-2571	4.47	0.0001						
	miR-77	3.86	0.0005	miR-311b	4.29	0.0001						
	miR-4197	−3.92	0.0004	miR-4142	4.02	0.0003						
	miR-236	−3.94	0.0004	mir-135a	3.98	0.0003						
	miR-2182	−4.03	0.0003	miR-8	3.97	0.0003						
	miR-758	−4.04	0.0003	miR-199b	3.85	0.0005						
	miR-982	−4.33	0.0001	miR-837	−3.90	0.0004						
	miR-34	−4.62	0.0001	miR-528	−4.03	0.0003						
	mir-4478	−4.73	<0.0001	miR-441a	−4.15	0.0002						
	miR-3479	−5.23	<0.0001	miR-395 g	−4.17	0.0002						
				mir-23a	−4.22	0.0002						
				miR-2357	−4.23	0.0002						
				mir-3673	−4.35	0.0001						
				mir-129	−4.64	<0.0001						
Area X												
	miR-2491	2.00	0.0002	miR-210	−1.96	<0.0001	miR-3751	1.35	<0.0001	miR-132 (6)	1.69 ± 0.06	0.0002
	miR-132 (2)	−1.79 ± 0.16	0.0002				miR-210 (4)	−1.71 ± 0.09	0.0003	miR-1713	−1.55	<0.0001

Fold changes are mean ± S.E.M. across all probes satisfying selection criteria, whereas *p*-value is that of the least significant result satisfying selection criteria

^a^Number of probes with expression meeting selection criteria in parentheses

**Fig. 3:**
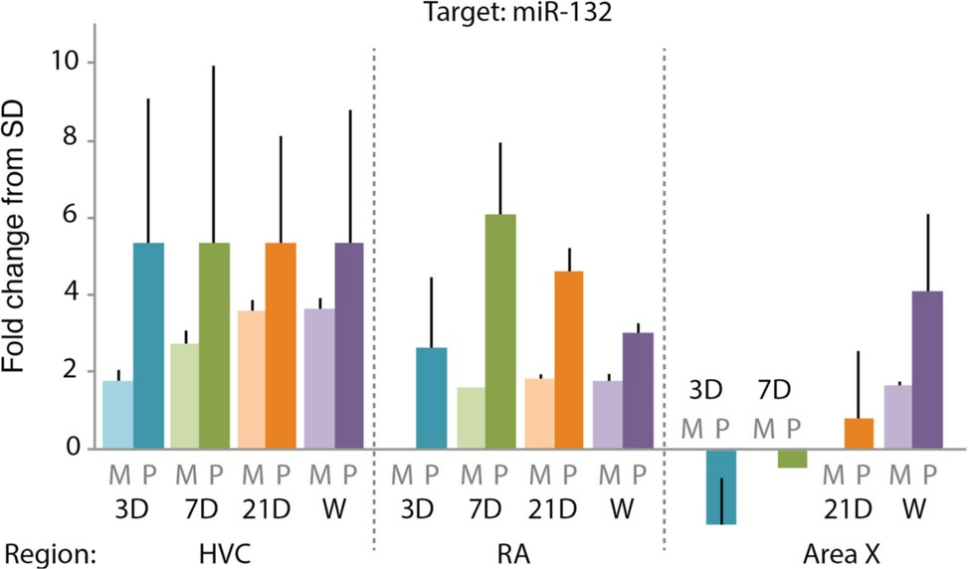
qRT-PCR validation miR microarray identification of miR-132 as differentially regulated between seasons. Fold change in expression of miR-132 from the microarray (M; *shown in light colors*) compared to qRT-PCR (P; *dark colors*). All fold-changes are relative to SD. miR-132 trended towards differential expression with qRT-PCR at LD + T 7D in HVC (adjusted-*p* = 0.0802) and achieved significant differential expression at LD + T 21D in RA (adjusted-*p* = 0.0427)

qRT-PCR of miR-132 on the same tissue used in the miR microarray validated the differential expression of miR-132 from the microarray analyses. The directional trends for miR-132 showed consistent up-regulation in qRT-PCR expression across all experimental groups for which miR-132 was significantly up-regulated in the microarray analyses (Fig. 3). Following FWER adjustment and Hommel correction for multiple comparisons, the differential miR-132 expression at LD + T 21D in RA achieved significance (adjusted-*p* = 0.0427).

Previous Illumina sequencing of auditory regions in zebra finches in response to song exposure identified 121 miRs conserved in other vertebrates and 34 novel miR sequences specific to zebra finches [52]. Consistent with this previous work we found that both conserved and bird-specific miRs were differentially expressed across seasons in the song control circuit. Some of the bird-specific miRs differentially expressed in HVC included miR-1562, −1782, −1915, −2962, −1700, −1575, and −1704 (Fig. 2b and Table 2).

Of the highly conserved miRs identified in this study, several are known to play roles in neural plasticity. For example, in the non-neurogenic nucleus RA, we found high expression of miR-135. Previous studies found that miR-135 inhibits the expression of the serotonin transporter and the inhibitory serotonin receptor, thereby promoting an increase in serotonin signaling [53]. The up-regulation of miR-135 in RA coincides with increases in spontaneous firing activity [38] and the probable increased serotonin signaling in RA [54, 55]. Another miR of interest up-regulated in RA during breeding conditions is miR-184. miR-184 regulates the translation of *NUMB* [51], a protein necessary for the survival of neural progenitor cells [56], a protein necessary for the survival of neural progenitor cells [57] and neuronal differentiation in the mammalian cortex [58]. miR-184 may confer low levels of NUMB in RA and prevent local neurogenesis. Finally, miR-129, down-regulated in RA during breeding conditions, has previously been shown to inhibit *FOXP2* translation [59], a protein thought to be associated with human speech [60], vocal learning in songbirds [61], and neurite outgrowth [62]. *FOXP2* has been found to be post-transcriptionally modified by miR-9 and miR-140, both of which are differentially expressed in zebra finch as a result of different social contexts for singing (i.e. directed towards a female or undirected) [63]. Moreover, the identification of avian gene targets of *FOXP2* through co-expression network analyses [51] suggests that *FOXP2* itself serves as a fulcrum for coordinating large regulatory gene networks. Given these previous findings and that miR-129, a predicted regulator of *FOXP2*, is differentially expressed across seasons in RA, the occurrence and importance of post-transcriptional regulation of *FOXP2* is of interest for additional exploration into regulatory networks related to song leaning and neural circuit plasticity.

In the neurogenic nucleus HVC, in addition miR-132, miR-212 expression also increased at 21D into LD + T. miR-212 transcription occurs at the miR-212/132 cluster [64]. Beyond functioning in tandem with miR-132, which will be discussed in greater detail below, miR-212 has also been shown to independently decrease glial cell proliferation [65]. Moreover, miR-212 expression in the rat dentate gyrus was increased following both acute and chronic electroconvulsive therapy [66], a therapy which increases neural stem cell proliferation [67] and increased neuronal fate specification [68]. Previous reports together with our data suggest that miR-212 functions both in tandem with miR-132 and independently to increase adult neurogenesis.

In HVC upon transition from LD + T to LDW, we found two miRs of interest that decreased in expression: miR-295 and miR-134. During LDW cell death peaks as HVC volume collapses [33] and neural stem cell proliferation increases significantly [35]. The timing of decreased miR-295 expression is consistent with a previous report that showed miR-295 suppressed autophagic cell death [69]: miR-295 down-regulation in HVC during LDW may permit the concurrent increase in HVC cell death. Additionally, we found that mir-134 expression was decreased in LDW compared to SD. miR-134 has been shown to regulate neural stem cell proliferation and neural plasticity, however, in a direction seemingly contradictory to our results. Decreased levels of miR-134 have been reported to increase synaptic plasticity and memory formation [70], dendritic spine formation and synaptic maturation [65], and neuronal survival [71]. The signaling mechanisms by which miR-134 exerts these effects, however, are just beginning to be elucidated.

### The seasonal interaction network of miR-132 in HVC

miR-132 mediates the integration of adult-born neurons in dentate gyrus [24], the arborization of new neurons in the hippocampus [23], and radial migration of neurons via expression of FOXP2 [72]. Based on the previously documented role of miR-132 in mammalian adult neurogenesis and synaptic formation and plasticity, and the fact that miR-132 was the most significantly up-regulated miR in HVC, we examined the miR–mRNA regulatory network of miR-132 more thoroughly. Prior to such analyses we first confirmed that the sequence of white-crowned sparrow miR-132 (i.e. zlg-mir-132) mature and seed regions was the same as the human miR-132 (hsa-mir-132) sequence (Additional file 1: Table S1 and Additional file 2: Figure S1). One-hundred percent identity is necessary because IPA software predicts targets based on the human miR seed region and mRNA 3‘UTR sequences. The zlg-mir-132 sequence was indeed 100 % identical to hsa-mir-132 in both the seed regions and the mature sequence. The full sequence of zlg-mir-132 was 62 % identical to the full sequence of hsa-mir-132 (miRBase MI0000449), 64 % to rno-mir-132 (rat; miRBase MI0000905), and 96 % to tgu-mir-132 (zebra finch; miRBase MI0016249). Generating a rooted phylogenetic tree with various vertebrate miR-132 sequences represented, grouped the zlg-miR-132 sequence with tgu-mir-132, further confirming correct full white-crowned sparrow sequence (Additional File 2: Figure S1).

**Fig. 4:**
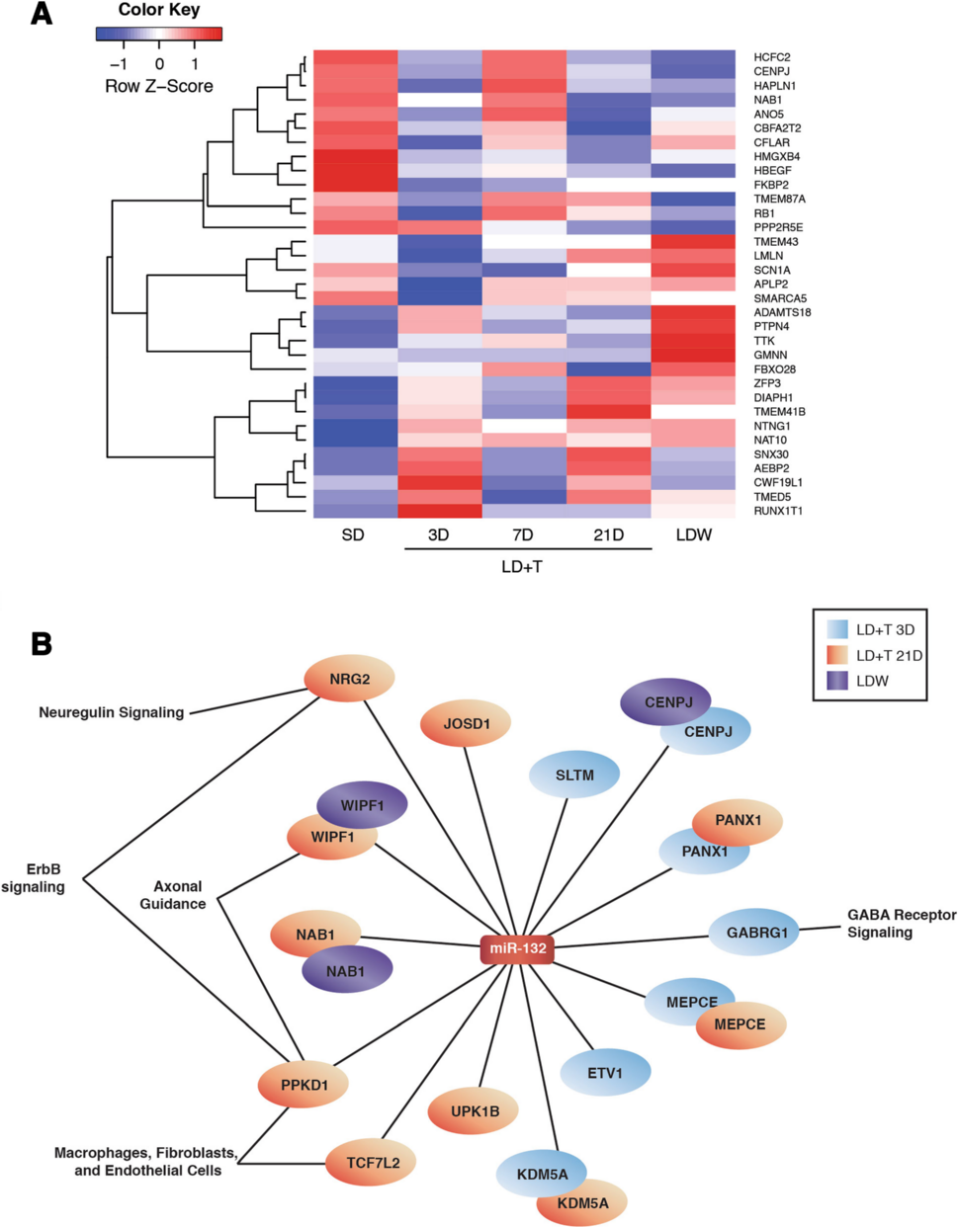
The seasonal miR-132–mRNA regulatory network in HVC. **a** The relative expression changes of mRNA targets of miR-132 that were differentially regulated in at least one experimental group (i.e. LD + T 3D, 21, or LDW) compared to SD, as well as having an inverse correlation to miR-132 in the same comparison. A *p*-value of <0.005 and a fold change >1.5 at any time point were used as selection criteria for mRNAs presented in the heat map. **b** An interaction network of mRNAs that were differentially anti-expressed with a fold change > -1.5 and *p* < 0.005 from conditions in which miR-132 was also differentially expressed (i.e. fold change > 2.0). IPA network analyses revealed several key pathways were down-regulated during periods of HVC new neuronal addition and functional incorporation

Through IPA software, miR-132 was predicted to target a total of 767 mRNAs. Thirty-three out of the 767 predicted mRNA targets were differentially expressed (>1.5 fold, *p* < 0.005) with expression inversely correlated with miR-132 between breeding and nonbreeding conditions in HVC (Fig. 4a). Of the thirty-three miR-132 targets eleven, two, fourteen, and five targets were differentially expressed during LD + T at 3D, 7D, 21D, and LDW, respectively (Fig. 4a). All of the predicted 33 seasonally-expressed targets of miR-132 were investigated for functional relatedness using IPA, from which we obtained the top significant canonical pathways represented by the filtered targets across all experimental groups (Table 3 and Fig. 4b). Independent analyses of functional relatedness of miR-132 targets from LD + T 3D, 7D, 21D, and LDW identified top canonical pathways including cell cycle control, PTEN signaling, calcium signaling, neuregulin signaling, and retinoic acid signaling (Table 4 and Fig. 4b).

**Table 3:** Top pathways for miR-132 and miR-210 predicted mRNA targets across all time points

Top Canonical Pathway		*p*-value	Ratio	Genes
miR-132 HVC				
	Molecular Mechanisms of Cancer	<0.0001	0.0301	E2F5, CFLAR, ADCY3, ARHGEF10, CDKN1A, MAPK1, TGFB2, PRKD1, RAP2B, PRKAG2, FOXO1
	RAR Activation	0.0001	0.0398	ADCY3, MAPK1, TGFB2, PRKD1, PRKAG2, NCOR1, GTF2H1
	VDR/RXR Activation	0.0001	0.0641	CDKN1A, TGFB2, PRKD1, NCOR1, FOXO1
	Breast Cancer Regulation by Stathmin1	0.0001	0.0366	E2F5, ADCY3, ARHGEF10, CDKN1A, MAPK1, PRKD1, PRKAG2
	ErbB Signaling	0.0001	0.0581	HBEGF, MAPK1, PRKD1, NRG2, FOXO1
	Pancreatic Adenocarcinoma Signaling	0.0003	0.0472	E2F5, HBEGF, CDKN1A, MAPK1, TGFB2
	Cell Cycle: G1/S Checkpoint Regulation	0.0005	0.0625	E2F5, CDKN1A, TGFB2, FOXO1
	PPARα/RXRα Activation	0.0005	0.0335	ADCY3, MAPK1, TGFB2, CAND1, PRKAG2, NCOR1
	Leptin Signaling	0.0008	0.0541	ADCY3, MAPK1, PRKAG2, FOXO1
	Acute Myeloid Leukemia Signaling	0.0009	0.0519	KITLG, MAPK1, TCF7L2, TCF7L1
miR-210 Area X				
	Phospholipase C Signaling	0.0022	0.0126	ADCY5, BTK, PPP1CB
	Dopamine Receptor Signaling	0.0033	0.0256	ADCY5, PPP1CB
	CDK5 Signaling	0.0052	0.0202	ADCY5, PPP1CB
	NAD Biosynthesis III	0.0066	0.1670	NMNAT2
	Phosphatidylcholine Biosynthesis I	0.0078	0.1430	PCYT1B
	NAD Salvage Pathway III	0.0078	0.1430	NMNAT2
	Cellular Effects of Sildenafil (Viagra)	0.0089	0.0155	ADCY5, PPP1CB
	β-adrenergic Signaling	0.0093	0.0150	ADCY5, PPP1CB
	Calcium Transport I	0.0100	0.1110	ATP2B3
	Dopamine-DARPP32 Feedback in cAMP Signaling	0.0135	0.0124	ADCY5, PPP1CB

**Table 4:** Top pathways for miR-132 in HVC predicted mRNA targets at each time point

Top Canonical Pathways		*p*-Value	Ratio	# Genes
LD + T 3D				
	Breast Cancer Regulation	0.0017	0.0209	4
	Estrogen-mediated S-phase Entry	0.0019	0.0833	2
	Molecular Mechanisms of Cancer	0.0028	0.0137	5
	Cell Cycle Checkpoint Control	0.0094	0.0364	2
	Wnt/β-catenin Sginaling	0.0103	0.0178	3
LD + T 7D				
	PTEN Signaling	0.0009	0.0254	3
	AMPK Signaling	0.0013	0.0224	3
	Melanoma Signaling	0.0030	0.0476	2
	Calcium Signaling	0.0029	0.0169	3
	Antiproliferative Role	0.0046	0.0317	2
LD + T 21D				
	ErbB Signaling	<0.0001	0.0581	5
	Neuregulin Signaling	0.0001	0.0455	4
	ErbB2/3 Signaling	0.0006	0.0526	3
	Molecular Mechanisms of Cancer	0.0006	0.0164	6
	ErbB4 Signaling	0.0007	0.0500	3
LDW				
	Melanocyte Development, Pigmentation Signaling	0.0001	0.0357	3
	CDK5 Signaling	0.0002	0.0303	3
	RAR Activation	0.0009	0.0170	3
	PPARα/RXRα Activation	0.0010	0.0168	3
	Pyridoxal 5’-phosphate Salvage Pathway	0.0023	0.0313	2

High levels of miR-132 expression in HVC are consistent with previous reports of high miR-132 expression in the brains of zebra finches [73]. Moreover, high expression of miR-132 in breeding condition HVC corroborates previous studies on the interactions between this miR, neurotrophins, and the ERK/MAPK signaling cascade. Transcription of miR-132 is controlled directly by the transcription factor cAMP-response element binding protein (CREB; [64]). Activation of CREB by phosphorylation occurs with BDNF binding the TRKB receptor [74], circadian gene oscillation [75], and synaptic activity via nuclear calcium signaling [76]. Activated CREB in combination with BDNF-mediated activation of the ERK/MAPK pathway in turn increases expression of miR-132 [77]. In HVC during breeding conditions, BDNF expression is enhanced by the presence of sex steroids [78] via the expansion of vasculature endothelial cells [79]. Concomitantly, CREB is co-expressed seasonally in HVC with androgen receptors [80], which trans-activate CREB when T binds [81]. Thus, in breeding conditions high T levels likely promote miR-132 expression via the enhanced activation of CREB and the production and signaling of BDNF in HVC. In turn, miR-132 represses the translation of a variety of repressor genes to promote cell cycle entry [82], neuronal addition [24], and arborization [23], and even the proliferation of endothelial cells [83] that secrete BDNF [79]. In this manner, mir-132 likely supports increased new neuronal addition and survival in HVC during breeding conditions.

The increase of miR-132 during breeding conditions driving a putative increase in BDNF, which in turn promotes HVC neuronal addition, is also consistent with previous studies. In both juvenile and adult birds, *BDNF* mRNA is expressed in in HVC and another song nucleus, LMAN, but not in Area X or RA [78, 84]. Moreover, expression in HVC is both higher during breeding conditions compared to nonbreeding conditions in white-crowned sparrows [78] and is necessary [85] and sufficient [86] for increased addition of adult-born neurons in HVC in canaries. *BDNF* expression also positively correlates with increased singing behavior, both of which positively correlate with the number and survival of new neurons added in HVC [87]. Alternatively, deafening (i.e., loss of auditory neural activity) decreases neuronal addition to HVC in adult zebra finches [88]. These studies highlight the seemingly inextricable link between BDNF, singing behavior, and addition of adult-born neurons in HVC, and suggest a common factor, such as miR-132, may be coordinating the expression of breeding condition neurotrophin expression, cytoarchitecture, and behavior.

### The seasonal interaction network of miR-210 in Area X

Of the significantly seasonally regulated miRs in Area X - another neurogenic and seasonally plastic song circuit nucleus - miR-210 stood out as a miR of interest. Given the role of miR-210 in promoting neural repair through angiogenesis [89–91], we investigated the miR–mRNA network of miR-210 more thoroughly. We first confirmed that the sequence of mature and seed regions of white-crowned sparrow miR-210 (i.e. zlg-mir-210) were 100 % identical to hsa-mir-210 sequence (Additional file 1: Table S1 and Additional file 3: Figure S2). The full sequence of zlg-mir-210 was 67 % identical to the full sequence of hsa-mir-210 (miRBase MI0000286), 67 % to rno-mir-210 (miRBase MI0000950), and 82 % to cli-mir-210 (pigeon; NCBI RefSeq NW_004973526.1). A rooted phylogenetic tree placed the zlg-miR-210 sequence in the same clade as several other bird species including the collard-fly catcher (*Ficedula albicollis*) and the pigeon (*Columbia livia*; Additional file 3: Figure S2).

**Fig. 5:**
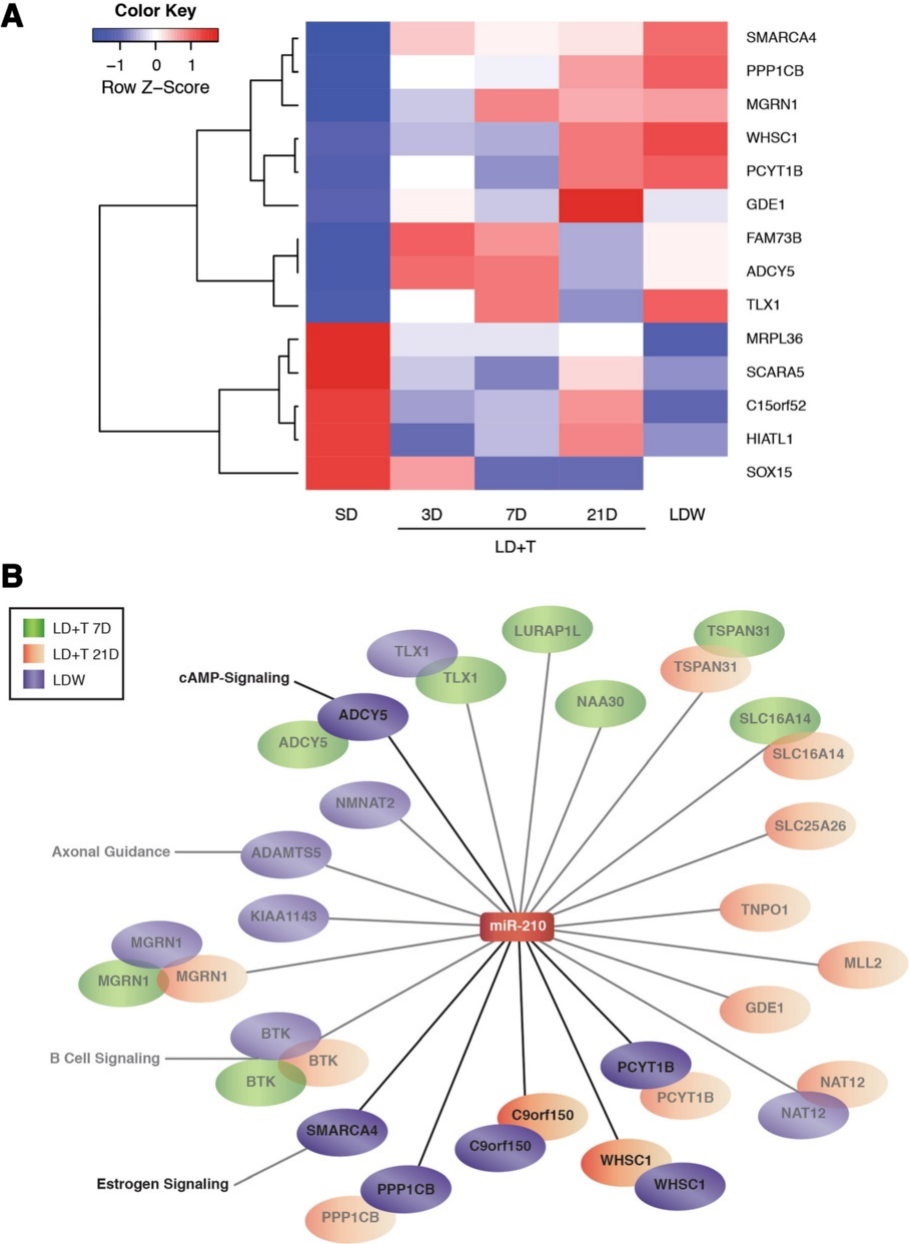
The seasonal miR-210 –mRNA regulatory network in HVC. **a** The expression fold changes of miR-210 mRNA that were differentially regulated and inversely correlated in at least one experimental group in which miR-210 was also differentially expressed (i.e. LD + T 7D, 21, or LDW) compared to SD. A *p*-value of <0.005 and fold change >1.5 at any time point were used as selection criteria for mRNAs presented in the heat map. **b** An interaction network of mRNAs that were differentially anti-expressed with a fold change > 1.5 and *p* < 0.005 (bright) or *p* < 0.05 (faded) from conditions in which miR-210 was also differentially expressed (i.e. fold change > –2.0). IPA network analyses revealed several key pathways were up-regulated in Area X during periods of volume expansion

Using IPA’s microRNA Target Prediction function we identified 1100 total targets of mir-210, 14 of which were differentially expressed (fold >1.5, *p* < 0.005) as assessed by mRNA microarray analysis and inversely correlated in Area X. Two predicted target mRNAs were differentially expressed in Area X between SD and LD + T at 21D, while six were differentially expressed between SD and LDW (Fig. 5a). Examining the filtered targets across experimental groups for functional relatedness identified top significant canonical pathways related to dopamine signaling, phospholipase signaling, and calcium signaling (Table 3). Independent analyses of functional relatedness of miR-210 targets from LD + T 7D, 21D, and LDW identified top canonical pathways including serotonin, GABA, and dopamine receptor signaling, calcium transport, and lymphocyte signaling (Table 5 and Fig. 5b).

**Table 5:** Top pathways for miR-210 in Area X predicted mRNA targets at each time point

Top Canonical Pathways		*p*-Value	Ratio	# Genes
LD + T 7D				
	Phospholipase C Signaling	0.0049	0.0084	2
	B Lymphocyte Signaling	0.0184	0.0244	1
	Serotonin Receptor Signaling	0.0197	0.0227	1
	Primary Immunodeficiency Signaling	0.0232	0.0192	1
	GABA Receptor Signaling	0.0299	0.0149	1
LD + T 21D				
	Phosphatidylcholine Biosynthesis	0.0039	0.1430	1
	Calcium Transport I	0.0050	0.1110	1
	Choline Biosynthesis III	0.0071	0.0769	1
	RAN Signaling	0.0093	0.0588	1
	Chemokine Signaling	0.0389	0.0141	1
LDW				
	Phospholipase C Signaling	0.0007	0.0126	3
	Dopamine Receptor Signaling	0.0015	0.0256	2
	CDK5 Signaling	0.0025	0.0202	2
	Cellular Effects of Sildenafil	0.0042	0.0155	2
	Cardiac β-adrenergic Signaling	0.0044	0.0150	2

Although the mechanism through which T or its metabolites reduces miR-210 expression is unclear, our data suggests that miR-210 is indeed under sex steroid control. No previous reports find evidence of direct control of miR-210 expression by sex steroids. Alternatively, miR-210 regulation by T may be indirect: miR-210 expression is induced by HIFα [92], which is suppressed by estrogen [93], a metabolite of T. Thus, increased systemic T, and its aromatization to estrogen, in Area X could prevent expression of HIF1α thereby also preventing mir-210 expression.

Previous work found that miR-210 expression was significantly increased in glioma tissue and inhibition of miR-210 promoted glial cell proliferation and glioma cell apoptosis [94]. Alternatively, miR-210 has also been shown to reduce the inflammatory release of cytokines [95], to promote VEGF [96, 97] and Notch 1 [91] expression, and to increase endothelial cell and neural precursor cell proliferation [90]. These seemingly contrasting roles for miR-210 remain to be reconciled. Given these reports and that we find both pro-neurogenic and anti-apoptotic genes predicted miR-210 targets are up-regulated during LD + T, miR-210 expression in the brain may represent a fine-scaled dose-dependent response to regulate levels of neural stem cell proliferation, neuronal versus glial fate specification, and cell survival.

## Conclusions

We identified two seasonal miR–mRNA interaction networks that likely coordinate the various processes and factors related to the integration of new neurons in neural circuits and for the seasonal plasticity of the HVC to RA and HVC to Area X neural pathways. Highly differentially expressed miRs including, miR-132 and miR-210, likely target many gene products that are also seasonally regulated. Our data are not only consistent with previous reports of miR signaling networks and roles, but also suggest that sex steroids may regulate the processes of seasonal plasticity via alterations in miR–mRNA expression networks. Herein, we provide opportunities to test novel genetic regulatory networks that control the diverse processes and mechanisms of functional incorporation of new neurons to the adult brain.

## Additional files

Additional file 1: Table S1. Complete genomic sequences of miRs of interest from Gambel’s white-crowned sparrow. (XLS 28 kb) Additional file 2: Figure S1. Confirmation of miR-132 sequence. A) Rooted phylogenetic tree based on the genomic miR-132 sequences of other closely related and distantlyrelated vertebrates. B) The genomic miR-132 sequence of white-crowned sparrows aligned with other species in ClustalW. The white-crowned sparrow miR-132 sequence was most similar to zebra finch miR-132 and more distantly related to mammalian miR-132 sequences. Asterisks indicate conservation of the given nucleotide across species. The mature region of the zlg-miR-132 sequence (highlighted in blue) had 100 % homology with the human sequence hsa-miR-132. (PDF 312 kb) Additional file 3: Figure S2. Confirmation of miR-210 sequence. A) Rooted phylogenetic tree based on the genomic miR-210 sequence of other closely related and distantlyrelated vertebrates. B) The genomic miR-210 sequence of white-crowned sparrows aligned with other species in ClustalW. The white-crowned sparrow miR-210 sequence was most similar to the pigeon, fly catcher, and penguin miR-210. Asterisks indicate conservation of the given nucleotide across species. The mature region of the zlg-miR-210 sequence (highlighted in blue) had 100 % homology with the human sequence hsa-miR-210. (PDF 325 kb) Additional file 4: ᅟ (XLSX 70 kb) Additional file 5: Table S3. Significantly differentially expressed miRNAs >2 fold. (XLS 33 kb)
