# Supplementary material for: Network analysis of microRNA and mRNA seasonal dynamics in a highly plastic sensorimotor neural circuit
Source: BMC Genomics. 2015 Nov 6;16:905. doi: 10.1186/s12864-015-2175-z (PMC4636775; doi:10.1186/s12864-015-2175-z)
Supplement: Additional file 2: Figure S1. — Confirmation of miR-132 sequence. A) Rooted phylogenetic tree based on the genomic miR-132 sequences of other closely related and distantlyrelated vertebrates. B) The genomic miR-132 sequence of white-crowned sparrows aligned with other species in ClustalW. The white-crowned sparrow miR-132 sequence was most similar to zebra finch miR-132 and more distantly related to mammalian miR-132 sequences. Asterisks indicate conservation of the given nucleotide across species. The mature region of the zlg-miR-132 sequence (highlighted in blue) had 100 % homology with the human sequence hsa-miR-132. (PDF 312 kb) [file 12864_2015_2175_MOESM2_ESM.pdf]

A

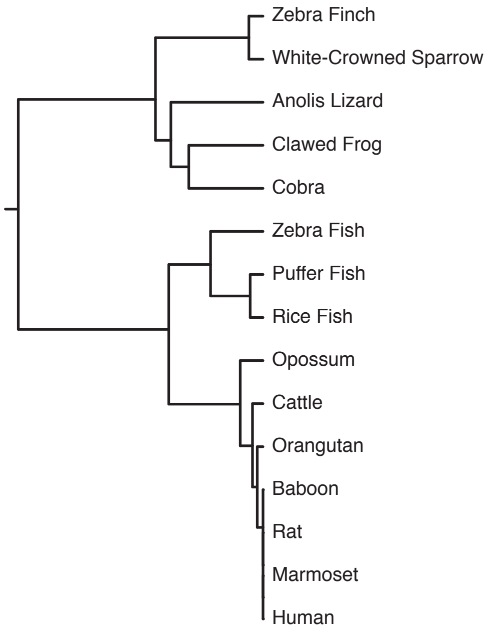

B

```
Callithrix jacchus        GCGCGTGGGCGTGCTGCGGGGCGACCATGGCTGTAGACTGTTACCTC-----CAGTTCCCACAGTAACAATCGAAAGCCACG-------------------------
Pongo pygmaeus            GCGCGTGGGCGTGCTGCGGGGCGACCATGGCTGTAGACTGTTACCTC-----CAGTTCCCACAGTAACAATCGAAAGCCACG-------------------------
Papio anubis              GCGCGTGGGCGTGCTGCGGGGCGACCATGGCTGTAGACTGTTACCTC-----CAGTTCCCACAGTAACAATCGAAAGCCACG-------------------------
Bos taurus                GCGCGTGGGCGTGCTGCGGGGCGACCATGGCTGTAGACTGTTACCTC-----CGGTTCCCACAGTAACAATCGAAAGCCACG-------------------------
Rattus norvegicus         GAGCGTGGGCGTGCTGCGGGGCGACCATGGCTGTAGACTGTTACCTC-----CGGTTCCCACAGTAACAATCGAAAGCCACG-------------------------
Monodelphis domestica     CCGCGTGGGCGTGCTGCGGGGCGACCATGGCTGTAGACTGTTACCCC-----TGGTTCCCACAGTAACAATCGAAAGCCACG-------------------------
Fugu rubripes             -----------TGCCCCCTAGCGACCATGGCTGTAGACTGTTACCACTGGTGCAGTTGCTACAGTAACAATCTAATGCCACG-------------------------
Oryzias latipes           -----------TGCCCCCTAGCGACCATGGCTGTAGACTGTTACCAGTGGTGCTGTTGCTACAGTAACAATCTAATGCCACG-------------------------
Danio rerio               -----------TGCCCCTTGGCGACCATGGCTGTAGACTGTTACCAC--GTGCCAACGCTACAGTAACAATCTAAAGCCACG-------------------------
Anolis carolinensis       -CACGGCAACCGTCTCCAGGGCAACCGTGGCTTTAGATTGTTACTGTGC-GGACGTGGGTTTGGTAACAGTCTACAGCCATGGTTGGTCCGGCGGACGCAA------
Ophiophagus hannah        --TCATCCTGCGTCTCCAGGGCAACCGTGGCTTTAGATTGTTACTGTGT-GGATGTGGGCT-GGTAACAGTCTACAGCCATGGTTGCCCGGACT-------------
Zonotrichia leucophyrus   -------------------GGCGACCGTGGCTTTAGATAGTTACTGTGC-CGCTGGGGG---AGTAACAGTCTACAGCCATGGTCGC--------------------
Taeniopygia guttata       ----ATCCGACGCCGCCCGGGCGACCGTGGCTTTAGATAGTTACTGTTC-CACTGGGGG---AGTAACAGTCTACAGCCATGGTCGCCGGGACAGGCAGACTCG---
Xenopus tropicalis        ---------CTGTCTCCAGGGCAACCGTGGCTTTAGATTGTTACTGTA---GTTCTGCATT-GGTAACAGTCTACAGCCATGGTCGCTCGGGCAAGATGC-------
Homo sapiens              -CCGCCCCCGCGTCTCCAGGGCAACCGTGGCTTTCGATTGTTACTGTG--GGAACTGGA---GGTAACAGTCTACAGCCATGGTCGCCCCGCAGCACGCCCACGCGC
                                              ** *** ***** * **  *****                   ****** ** *  **** *
```

Figure S1. Confirmation of miR-132 sequence. A) Rooted phylogenetic tree based on the genomic miR-132 sequences of other closely related and distantly-related vertebrates. B) The genomic miR-132 sequence of white-crowned sparrows aligned with other species in ClustalW. The white-crowned sparrow miR-132 sequence was most similar to zebra finch miR-132 and more distantly related to mammalian miR-132 sequences. Asterisks indicate conservation of the given nucleotide across species. The mature region of the zlg-miR-132 sequence (highlighted in blue) had 100% homology with the human sequence hsa-miR-132.
