# Supplementary material for: Network analysis of microRNA and mRNA seasonal dynamics in a highly plastic sensorimotor neural circuit
Source: BMC Genomics. 2015 Nov 6;16:905. doi: 10.1186/s12864-015-2175-z (PMC4636775; doi:10.1186/s12864-015-2175-z)
Supplement: Additional file 3: Figure S2. — Confirmation of miR-210 sequence. A) Rooted phylogenetic tree based on the genomic miR-210 sequence of other closely related and distantlyrelated vertebrates. B) The genomic miR-210 sequence of white-crowned sparrows aligned with other species in ClustalW. The white-crowned sparrow miR-210 sequence was most similar to the pigeon, fly catcher, and penguin miR-210. Asterisks indicate conservation of the given nucleotide across species. The mature region of the zlg-miR-210 sequence (highlighted in blue) had 100 % homology with the human sequence hsa-miR-210. (PDF 325 kb) [file 12864_2015_2175_MOESM3_ESM.pdf]

A

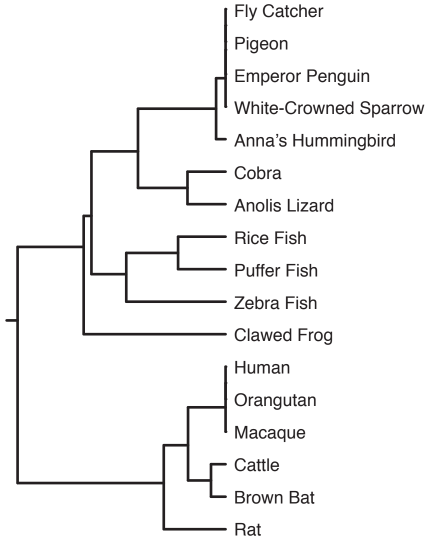

B

```
Anolis carolinensis        CCAGTTCTCCAGGAGCAGATG-AGCCACTGACT-AACGCACATTGTGCTTCT--CGTGTCCCCAC-TGTGC-GTGTGACAGCGGCTAACCTGCTTTTCGGAC---------------------
Ophiophagus hannah         ---------CAGAAGCAGGTG-AGCCACTGACT-AACGCACATTGTGCTGCT---ATGTCCCCAC-TGTGC-GTGTGACAGCGGCTAACCTGC------------------------------
Fugu rubripes              -------TCTAAAAGCAGGTG-AGCCACTGACT-AACGCACATTGCGCCAGTT-GACAGTTCCAC-TGTGC-GTGTGACAGCGGCTAACCTG-------------------------------
Oryzias latipes            TCCGAGTTCTAAAAGCAGGTA-AGCCACTGACT-AACGCACATTGTGCGTGT--GACAGATCCAC-TGTGC-GTGTGACAGCGGCTAACCTG-------------------------------
Danio rerio                --------------GCAGGTA-AGCCACTGACT-AACGCACATTGCGCCTATT-CTCCACTCCAC-TGTGC-GTGTGACAGCGGCTAACCAG-------------------------------
Xenopus tropicalis         -------TCCAGATGCAGGTC-AGCCACTGACT-AACGCACATTGCGCTGCTCCTAAAATGCCAC-TGTGC-GTGTGACAGCGGCTAACCAGCATCTAGGAA---------------------
Columba livia              -----CCTCCAGGAGCAGGTG-AGCCACTGACT-AACGCACATTGTGCTCTC--GGCGACTCCAC-TGTGC-GTGTGACAGCGGCTA------------------------------------
Zonotrichia leucophyrus    -----CCTCCAGGAGCAGGTG-AGCCACTGACT-AACGCACATTGTGCTCTC--GGCGACTCCAC-TGTGC-GTGTGACAGCGGCT-------------------------------------
Aptenodytes forsteri       -----CCTCCAGGAGCAGGTG-AGCCACTGACT-AACGCACATTGTGCTCTC--GGCGACTCCAC-TGTGC-GTGTGACAGCGGCT-------------------------------------
Ficedula albicollis        -----CCTCCAGGAGCAGGTG-AGCCACTGACT-AACGCACATTGTGCTCTC--GGCGACTCCAC-TGTGC-GTGTGACAGCGGCT-------------------------------------
Calypte anna               -----CCTCCAGGAGCAGGTG-AGCCACTGACT-AACGCACATTGTGCTCTC--GGTGACTCCAC-TGTGC-GTGTGACAGCGGCT-------------------------------------
Homo sapiens               -----CCTCCAGGCGCAGGGC-AGCCCCTGCCC-ACCGCACACTGCGCTGCCCC--AGACC-CAC-TGTGC-GTGTGACAGCGGCTGATCTGTGCCTG-GGCAGCGCGACCC-----------
Pongo pygmaeus             -----CCTCCAGGCGCAGGGCCAGCCCCTGCCCCACCGCACACTGCGCTGCCCCCAAGACCACACGTGTGCCGTGTGACAGCGGCTGATCTGTGCCTGAGGCAGCGCGACCC-----------
Macaca mulatta             -----CCTCCAGGCGCAGGGC-AGCCCCTGCCC-ACCGCACACTGCGCTGCCCC--AGACC-CAC-TGTGC-GTGTGACAGCGGCTGATCTGTGCCTG-GGCAGCGCGACCC-----------
Bos taurus                 -----CCTCCAGGCGCAGGGC-AGCCACTGCCC-ACCGCACACTGCGCTGCTCC--GGACC-CAC-TGTGC-GTGTGACAGCGGCTGATCTGTCCCTG-GGCAGCGCGACC------------
Rattus norvegicus          -----CCTCCAGGCTCAGGAC-AGCCACTGCCC-ACAGCACACTGCGTTGCTCC--GGACC-CAC-TGTGC-GTGTGACAGCGGCTGATCTGTCCCTG-GGCAGCGCGAACC-----------
Eptesicus fuscus           -----CCTCCAGGCGCAGGGC-AGCCACTGCCC-ACCGCACACTGCGCTGCTCC--GGACC-CAC-TGTGC-GTGTGACAGCGGCTGATCTGCTCCTG-GGCAGCGCGACCGCCCCGGCCCCT
                                          ***    **** *** *  *  ***** ** *               *** ***** **************
```

Figure S2. Confirmation of miR-210 sequence. A) Rooted phylogenetic tree based on the genomic miR-210 sequence of other closely related and distantly-related vertebrates. B) The genomic miR-210 sequence of white-crowned sparrows aligned with other species in ClustalW. The white-crowned sparrow miR-210 sequence was most similar to the pigeon, fly catcher, and penguin miR-210. Asterisks indicate conservation of the given nucleotide across species. The mature region of the zlg-miR-210 sequence (highlighted in blue) had 100% homology with the human sequence hsa-miR-210.
